# Neurobiological Mechanisms of Vitamin D in Anxiety: Evidence from Rodent Models and Bioinformatics-Based Target Identification

**DOI:** 10.3390/nu18162707

**Published:** 2026-08-19

**Authors:** Alice S. Medeiros, Sofia A. Rodrigues, Ana Lúcia S. Rodrigues

**Affiliations:** Department of Biochemistry, Center of Biological Sciences, Federal University of Santa Catarina, Florianópolis 88037-000, SC, Brazil; alicemedeiross582@gmail.com (A.S.M.); sofiaalvesrodrigues10@gmail.com (S.A.R.)

**Keywords:** anxiety disorders, vitamin D, rodent models, bioinformatics

## Abstract

**Background/Objectives**: Anxiety disorders represent a major global public health challenge, with increasing prevalence and substantial unmet therapeutic needs. Emerging evidence suggests that vitamin D modulates multiple neurobiological pathways involved in anxiety, although the underlying mechanisms remain incompletely understood. This review provides a comprehensive synthesis of experimental and bioinformatics evidence on the anxiolytic effects of vitamin D. **Methods**: The current literature on the anxiolytic effects of vitamin D in rodent models was critically reviewed, focusing on evidence related to neurotransmitter systems, neuroplasticity, neurotrophic factors, neuroinflammation, oxidative stress, and receptor-mediated signaling. Bioinformatics analyses were incorporated to identify molecular targets and signaling pathways potentially linking vitamin D to anxiety-related mechanisms. **Results**: The integrated evidence indicates that vitamin D exerts pleiotropic neurobiological effects through the coordinated modulation of neuroinflammatory signaling, neuroplasticity, monoaminergic neurotransmission, neuroendocrine regulation, epigenetic mechanisms, and vitamin D metabolism, all of which are implicated in the pathophysiology of anxiety disorders. Bioinformatics analyses further supported these findings by identifying molecular targets and signaling pathways potentially involved in the anxiolytic effects of vitamin D. **Conclusions**: Current evidence supports a potential role for vitamin D in anxiety disorders, but further translational and clinical studies are required to establish its therapeutic value.

## 1. Introduction

Anxiety disorders are among the most prevalent mental health conditions worldwide, affecting millions of individuals and often leading to substantial impairment in daily functioning, social interactions, and overall quality of life. Recent estimates from the Global Burden of Disease Study 2019 indicate that the prevalence, incidence, and years lived with disability associated with mental disorders increased significantly between 2019 and 2021, with anxiety and depressive disorders accounting for the largest increases [1]. Anxiety disorders comprise a heterogeneous group of psychiatric disorders characterized by excessive fear, anxiety, and related behavioral disturbances that are disproportionate to the actual threat or persist beyond developmentally appropriate periods. According to the Diagnostic and Statistical Manual of Mental Disorders, Fifth Edition (DSM-5), this category includes separation anxiety disorder, selective mutism, specific phobia, social anxiety disorder (social phobia), panic disorder, agoraphobia, generalized anxiety disorder, substance/medication-induced anxiety disorder, anxiety disorder due to another medical condition, and other specified and unspecified anxiety disorders. Although these disorders share common features, they differ in their underlying triggers, clinical presentation, and diagnostic criteria [2].

Neurotransmitter systems play a central role in the regulation of emotional processing, stress responsiveness, and anxiety-related behaviors. Alterations in the brain levels and signaling of monoamines, including serotonin (5-HT), norepinephrine (NE), and dopamine (DA), have been consistently implicated in the pathophysiology of anxiety disorders [3,4].

Consistent with the importance of monoaminergic signaling, a randomized clinical trial demonstrated that supplementation with Lactobacillus plantarum DR7 alleviated anxiety symptoms in stressed adults while modulating serotonergic and dopaminergic pathways and reducing cortisol and pro-inflammatory cytokine levels, highlighting the contribution of the gut–brain axis to neurotransmitter homeostasis [5]. Furthermore, activation of the microglial purinergic receptor P2X7 (P2X7R) has emerged as another mechanism contributing to anxiety, as chronic stress facilitates P2X7R signaling, leading to NLRP3 inflammasome activation, neuroinflammation, and anxiety-like behavior, while pharmacological stabilization of its endogenous regulator, Na+/K+-ATPase α1 (NKAα1), reverses these effects [6]. In addition, an imbalance between excitatory glutamatergic and inhibitory GABAergic neurotransmission is considered a key mechanism underlying anxiety, as excessive glutamatergic activity or impaired GABAergic inhibition can promote neuronal hyperexcitability and dysfunctional fear responses [7].

Cholinergic signaling has also emerged as an important modulator of anxiety, as the ability of acetylcholine to coordinate neuronal network activity across multiple brain regions is essential for regulating attention, arousal, and stress-responsive neural circuit [8], and has been implicated in regulating neurogenesis [9]. Supporting the contribution of neurotransmitter imbalance to anxiety, elevated brain levels of the trace amine β-phenylethylamine resulting from MAO-B deficiency have been associated with reduced anxiety-like behaviors in mice [10].

Collectively, these findings highlight that dysregulation of multiple neurotransmitter systems is closely associated with neuroplasticity, oxidative stress, excitotoxicity, neuroinflammation that contributes to anxiety pathophysiology [7]. These interconnected processes culminate in reduced BDNF signaling, dendritic remodeling, synaptic loss, hippocampal and prefrontal cortical atrophy, and increased amygdala activity [7].

Accumulating evidence indicates that impaired neuroplasticity plays a central role in the pathophysiology of anxiety disorders. Particularly, selective impairment of adult hippocampal neurogenesis in transgenic animals has been associated with a marked increase in anxiety-like behaviors [11]. Brain-derived neurotrophic factor (BDNF) is a critical regulator of neuronal survival, synaptic plasticity, and activity-dependent synaptic remodeling, processes that are essential for adaptive responses to environmental challenges. Impairments in BDNF signaling have been consistently associated with maladaptive neuroplasticity and increased vulnerability to anxiety disorders [12]. In this context, the common BDNF Val66Met polymorphism, which disrupts activity-dependent BDNF secretion without affecting its overall expression, has provided important evidence linking neuroplasticity to anxiety-related behaviors. Mice carrying the Met allele exhibit heightened anxiety-like behavior in response to stress and reduced responsiveness to the anxiolytic effects of fluoxetine, suggesting that impaired BDNF-dependent synaptic plasticity may compromise neural circuit adaptations required for stress resilience [13]. Furthermore, chronic stress has been shown to impair BDNF-dependent synaptic function even in the absence of changes in total BDNF expression, attenuating BDNF-induced glutamate release in the prefrontal cortex while promoting anxiety- and depression-like behaviors, suggesting that functional deficits in BDNF signaling, rather than changes in its expression alone, may critically contribute to stress-related psychopathology [14]. Moreover, chronic stress has been shown to induce epigenetic alterations associated with the downregulation of multiple Bdnf transcripts in the prefrontal cortex of mice, while pharmacological inhibition of DNA methylation attenuated anxiety-like behavior in environmentally enriched mice, highlighting the contribution of epigenetic regulation of BDNF-dependent neuroplasticity to stress susceptibility [15]. Altogether, these findings support the concept that alterations in BDNF-mediated neuroplasticity contribute to the pathophysiology of anxiety disorders. Consequently, strategies aimed at restoring neuroplasticity and enhancing BDNF signaling are increasingly recognized as promising therapeutic approaches for anxiety disorders.

Anxiety disorders are primarily mediated by the threat-fear neural circuit, which comprises interconnected regions including the prefrontal cortex, amygdala, hippocampus, anterior cingulate cortex, and insula that regulate threat perception, fear processing, and emotional responses. Increasing evidence from both preclinical and clinical studies indicates that neuroinflammation within these brain regions contributes to circuit dysfunction and the development of anxiety symptoms [16].

A growing body of evidence indicates that chronic low-grade inflammation, characterized by increased circulating pro-inflammatory cytokines and activation of immune pathways, can disrupt brain function by altering neurotransmitter systems, impairing neuroplasticity, and promoting dysregulation of stress-responsive neural circuits. Peripheral inflammatory mediators may also communicate with the central nervous system through multiple pathways, including disruption of blood–brain barrier integrity, activation of vagal afferents, and recruitment of immune cells, ultimately contributing to microglial activation and sustained neuroinflammatory responses. These processes have been implicated in the development and persistence of anxiety symptoms [17]. Supporting this hypothesis, a large prospective cohort study using data from the UK Biobank demonstrated that metabolic syndrome (MetS) was associated with a significantly increased risk of developing anxiety over a mean follow-up of 12 years. The risk increased progressively with the number of MetS components, indicating a dose-dependent relationship, and was particularly pronounced in individuals younger than 55 years. Importantly, mediation analyses revealed that systemic inflammatory markers, including C-reactive protein, leukocytes, neutrophils, lymphocytes, platelets, and the INFLA score, partially explained the association between MetS and anxiety, with inflammatory factors collectively accounting for approximately one-third of the observed effect. These findings provide strong epidemiological evidence that chronic low-grade inflammation is a key mechanism linking metabolic dysfunction to the development of anxiety disorders [18].

In addition to neuroinflammation, oxidative stress is now widely recognized as a significant contributor to the pathophysiology of anxiety disorders. The two processes are closely interconnected: chronic inflammation promotes the generation of reactive oxygen species, and oxidative stress further amplifies inflammatory signaling, creating a self-perpetuating cycle. Evidence from preclinical and clinical studies indicates that this neuroinflammatory-oxidative imbalance is associated with anxiety symptoms [19,20]. Moreover, clinical studies have consistently reported increased levels of oxidative stress biomarkers, together with markers of DNA damage (e.g., 8-OHdG), and impaired antioxidant defenses in individuals with anxiety symptoms or diagnosed anxiety disorders, reinforcing the role of oxidative stress in anxiety pathophysiology [21,22]. These findings highlight oxidative stress as a potential therapeutic target and support the investigation of antioxidant-based strategies for the prevention and treatment of anxiety disorders.

Due to the multifactorial and complex nature of anxiety disorders, their treatment remains a significant clinical challenge. Current therapeutic approaches include pharmacological interventions, such as selective serotonin reuptake inhibitors (SSRIs), serotonin–norepinephrine reuptake inhibitors (SNRIs), and benzodiazepines, as well as psychological interventions [23]. While these treatments are effective for many patients, a significant number experience inadequate symptom relief, delayed therapeutic response, treatment resistance, or adverse effects that hinder long-term adherence [24]. Consequently, there is a growing interest in identifying new interventions that target the underlying biological mechanisms of anxiety disorders to improve treatment outcomes and reduce their burden.

Vitamin D has emerged as a promising anxiolytic candidate due to its potential role in modulating neurotransmission, neuroplasticity, neuroinflammation, and oxidative stress [25], key mechanisms involved in the pathophysiology of anxiety disorders. Vitamin D exerts pleiotropic effects by modulating multiple molecular targets across different cell populations, including neurons and glial cells. Within the central nervous system, its biological actions are mediated through genomic and non-genomic signaling pathways involving the vitamin D receptor (VDR), thereby contributing to the maintenance of neural homeostasis [26].

Concurrently, vitamin D deficiency has become a global public health concern, affecting diverse populations including women, older adults, and individuals with limited sunlight exposure [27]. This growing prevalence has generated considerable interest in the potential impact of vitamin D status on mental health outcomes, particularly anxiety.

In humans, growing evidence suggests a relationship between vitamin D status and anxiety. Low circulating 25-hydroxyvitamin D levels have been associated with an increased prevalence and severity of anxiety symptoms [28], while clinical studies have reported mixed findings regarding the anxiolytic effects of vitamin D supplementation [29,30]. Overall, these observations support the potential involvement of vitamin D in the pathophysiology of anxiety disorders.

While recent network pharmacology studies have begun to elucidate the molecular mechanisms linking calcitriol to anxiety [31], whether these predicted pathways are consistent with the preclinical evidence obtained using vitamin D3 (cholecalciferol) remains largely unexplored. Since vitamin D3 is the form most frequently administered in experimental rodent studies, integrating network pharmacology with findings from animal models may provide a more comprehensive understanding of the mechanisms potentially underlying its anxiolytic-like effects and anxiety-related behavioral outcomes. Accordingly, this review summarizes the preclinical evidence on vitamin D3 in anxiety and extends previous bioinformatic investigations by identifying molecular networks and signaling pathways associated with its potential therapeutic actions.

## 2. Materials and Methods

This study presents a narrative review of the scientific literature to summarize, analyze, and discuss the available evidence on the anxiolytic effects of vitamin D in rodent models, highlighting the biological mechanisms that may account for these effects.

The literature search was conducted using PubMed, with no date restrictions applied. The search covered the available literature from database inception to 17 July 2026. The following descriptors were used, either individually or in combination: cholecalciferol, vitamin D3, 1,25-D3, calcitriol, vitamin D receptor, anxiety, anxiety disorders, mice, rat, central nervous system, hippocampus, cortex, neuroinflammation, neuroplasticity, oxidative stress, neurotransmitter, monoamine. ScienceDirect and the MDPI journal platform were additionally consulted to locate relevant publications and obtain the full-text versions of articles considered pertinent to the scope of the review. Relevant MDPI articles were identified through PubMed and were not treated as a separate database.

Studies were considered eligible if they investigated the effects of vitamin D3 (cholecalciferol), or related vitamin D metabolites on anxiety-related outcomes in rodent models and/or addressed biological mechanisms potentially underlying these effects. Studies were excluded if they were outside the scope of the review or did not provide relevant experimental or mechanistic information regarding the relationship between vitamin D and anxiety-related outcomes.

Given the narrative nature of this review, no formal assessment of risk of bias or quantitative synthesis was undertaken. Instead, the included studies were evaluated based on the consistency of their findings, the biological plausibility of the proposed mechanisms, and their contribution to an integrated understanding of the role of vitamin D in anxiety-related outcomes.

For the bioinformatic analyses, keywords were used to search two databases for target identification: the Comparative Toxicogenomics Database (CTD) (https://ctdbase.org/) and UniProt (https://www.uniprot.org/). CTD provides comprehensive coverage of species and includes more than just human data. It enables users to search for interactions between chemical compounds and associated genes in several diseases and contains both curated data and inferred data from selected chemical–gene interactions [32]. UniProt is recognized as the main protein database, covering virtually all animal species for which genomic data is available [33]. In the CTD, no species restriction was applied. For anxiety, only curated data were included, and only disorders classified as anxiety disorders according to the DSM-5 were considered. In UniProt, searches were performed separately for *Mus musculus* and *Rattus norvegicus*.

Duplicate removal and target overlap analyses were performed using Venny v.2.1.0 (https://bioinfogp.cnb.csic.es/tools/venny/, accessed on 15 August 2026). After identifying the shared targets, CTD-derived targets were cross-referenced according to species to verify their presence in *Mus musculus* and *Rattus norvegicus*. Because the vitamin D receptor (Vdr) was not identified among the overlapping targets, it was manually included based on the evidence summarized in Table 1.

The final list of molecular targets was imported into the STRING v.12.0 (https://string-db.org/) database to construct species-specific protein–protein interaction (PPI) networks for mice and rats. High-confidence interaction parameters (confidence score = 0.700) and network expansion by 40 additional interactors were applied. The resulting PPI networks were subsequently imported into Cytoscape v.3.10.4 for topological and statistical analyses. Rather than prioritizing network nodes solely according to conventional topological metrics, such as degree or betweenness centrality, we adopted a biologically informed approach that considered evidence of expression and specificity in the nervous system. This approach was motivated by evidence that network hubs can differ substantially in their tissue-expression breadth and functional roles, and that tissue-specific hubs may represent biologically distinct entities from broadly expressed, highly connected proteins [34]. Therefore, nervous system expression and tissue specificity were considered as complementary criteria for assessing the biological relevance of network nodes in the present study.

To further characterize the biological relevance of the prioritized network targets, Gene Ontology (GO) and Kyoto Encyclopedia of Genes and Genomes (KEGG) enrichment analyses were performed in ShinyGo v.0.85.1 (South Dakota State University, Brookings, USA) (https://bioinformatics.sdstate.edu/go/, accessed on 15 August 2026) to characterize the biological processes, molecular functions, cellular components, and signaling pathways associated with the identified proteins [35]. A false discovery rate (FDR) < 0.05 was used as the significance threshold. Fold enrichment was used to compare the background frequency of all genes annotated with a given term in a specific species with the sample frequency, representing the number of genes in the analysis that fall under the same term. KEGG pathways and GO terms with the lowest FDR values and highest fold enrichment values were prioritized to identify the most significantly enriched pathways and biological functions.

## 3. Anxiolytic Effects of Vitamin D: Preclinical Evidence

This section provides an overview of the preclinical evidence from experimental studies in rodents investigating the potential anxiolytic effects of vitamin D and the possible underlying neurobiological mechanisms. The main findings of these studies are summarized in Table 1.

**Table 1:** Behavioral and biochemical effects of vitamin D in experimental models of anxiety.

Reference	Biochemical Findings	Behavioral Outcomes	Vitamin D Treatment	Sex/Specie/Strain/Age
[36]	No direct biochemical data; Possible altered cholinergic/ catecholaminergic signaling	VdrKO: ↑ anxiety-like behavior; ↓ exploration; ↑ grooming; ↑ EPM latency; ↓ light-zone exploration	Genetic ablation of vitamin D receptors (VDR knockout model); no vitamin D supplementation	Male, 129 strains mice, 24–30 weeks old; VDR knockout (−/−), wild-type (+/+) and heterozygous (+/−)
[37]	No direct biochemical data; Possible DA/ACh, Ca^2+^ and brain-region involvement	VdrKO: ↑ grooming in ACT, OF, EPM and HR; No major changes in locomotion, olfaction or anxiety-like behavior	Genetic ablation of vitamin D receptors (VDR knockout model); no vitamin D supplementation	Male, 129/S1 strains mice, 24–30 weeks old; VDR knockout (−/−) and wild-type (+/+) mice
[38]	Maternal VD3 intake changed offspring serum 25(OH)D3 and Ca; excessive VD3 ↓ body length, body and brain weight	Juveniles: ↑ anxiety-like behavior in EPM, with ↑ grooming and ↓ OA entries; Adult anxiety effects mostly absent	Maternal diets containing 0–10 IU/g VD3 from mating until weaning (PND 18)	Male and female Sprague–Dawley offspring, tested at PND 35–40 and PND 100–105
[39]	No direct biochemical data; Possible involvement of VDRs, 5-HT, DA, NE, GABA and estrogen signaling	VD3 1.0/2.5 mg/kg: ↓ anxiety-like behavior in EPM and LDT; 17β-estradiol potentiated anxiolytic-like effects; VD3 5.0 mg/kg: ↑ anxiety-like behavior	VD3 (1.0, 2.5, or 5.0 mg/kg SC for 14 days), alone or with 17β-estradiol; diazepam was the comparator	Adult female Wistar rats; long-term ovariectomy model
[40]	↑ hippocampal DA and 5-HT; ↓ 5-HIAA/5-HT; ↑ DOPAC/HVA	High-dose VD3 ↓ anxiety-like behavior in EPM and LDT; 17β-estradiol potentiated effects; ↑ grooming in OFT	VD3 (1.0, 2.5, or 5.0 mg/kg SC for 14 days), alone or with 17β-estradiol; diazepam was the positive control	Adult female Wistar rats; ovariectomized model of estrogen deficiency
[41]	Altered hippocampal plasticity/activity genes: BDNF, CREB, Arc, Egr-1 and SNAP25; no major anatomical changes	No anxiety/depression-like changes; overdose transiently ↑ locomotion; VD deficiency ↓ spatial learning; deficiency/overdose↓ long-term memory	Postnatal diets containing 0, 1000, or 10,000 IU VD3; male offspring continued the same diet after weaning	Male C57BL/6 mice; postnatal model evaluated at 6 and 17 weeks of age
[42]	OVX ↓ estradiol and 25(OH)VD3; vitamin D3 ↑ circulating 25(OH)VD3 and estradiol; Ca unchanged	VD3 ↓ anxiety-like behavior in EPM and LDT across doses; VD3 + 17β-estradiol showed stronger effects than estradiol alone	VD3 (1.0, 2.5, or 5.0 mg/kg/day SC for 14 days), alone or with 17β-estradiol	Female Wistar rats, 16–18 months old; ovariectomized (OVX) for 12 weeks to induce long-term estrogen deficiency
[43]	UCMS ↑ MDA, IL-6 and corticosterone; ↓ thiols, SOD and CAT; vitamin D reversed these changes	Vitamin D improved UCMS-induced behavior in OFT, EPM and FST; ↑ center/OA time; ↓ immobility	VD3 administered intraperitoneally at 100, 1000, or 10,000 IU/kg during UCMS exposure	Adult male Wistar rats, (exposed to UCMS for 4 weeks)
[44]	↑ serum 25(OH)VD3; ↓ hippocampal NR2A/GluN2A mRNA/protein; no α7 nAChR effect	VD3 attenuated nicotine withdrawal-induced anxiety; ↑ center time in OF; ↓ marble burying	VD3-enriched diet (10,000 IU/kg for 6 weeks) during nicotine exposure; anxiety assessed during spontaneous withdrawal	Male C57BL/6 mice, 9/10 weeks old
[45]	Altered CYP27A1, CYP27B1 and CYP24A1 expression; supplementation normalized neuronal, synaptic, GABA receptor and NF-κB-related genes	Vitamin D supplementation ↓ anxiety-like behavior in OF and partially improved rotarod performance	1 IU/g (control diet), 10 IU/g (supplemented diet) or 0.1 IU/g (deficient diet) VD3	Female Mecp2+/− C57BL/6 mice, 4 weeks old (behavioral testing at 3, 5, and 7 months)
[46]	↑ serum TNF-α and IL-1β; ↓ PFC BDNF and VDR; Supplementation/exercise reversed these changes	Maternal vitamin D deficiency: ↓ exploration in EPM/OFT, ↑ grooming, ↑ immobility, ↓ diving; Supplementation/exercise improved behavior	Standard (1000 IU/kg) or vitamin D_3_-deficient diet (0 IU/kg), with or without treadmill exercise	Wistar rats; female dams (3 weeks old), and adult male offspring evaluated at PND90
[47]	↓ blood glucose, ROS and TBARS; modulated purinergic signaling; ↑ ectonucleotidase activity; regulated P2X7, A1 and A2A receptors	VD3 improved anxiety-like behavior and NOR memory; ↑ novel-object exploration and recognition index	VD3 (90 μg/kg/day, gavage for 30 days), with or without metformin (400 mg/kg/day), in STZ-induced T1DM rats	Male Wistar rats, 60 days old
[48]	Corticosterone unchanged; limited biochemical/molecular analyses	Gestational vitamin D improved maternal care; Prevented DEX-induced anxiety-like behaviors in offspring; Improved memory mainly in males	VD3 (500 IU/day, oral gavage, entire pregnancy), with or without DEX (0.1 mg/kg, gestational days 14–19)	Pregnant 3-month-old Wistar rats; male and female offspring assessed during lactation and at PNM 3, 6, and 12
[49]	↑ serum 25(OH)D3; ↑ hippocampal DCX; no ↑ apoptosis	Vitamin D ↓ anxiety-like behavior in EPM; calcium ↑ locomotion/exploration and object recognition memory	VD3 (400 IU/kg, gavage for 4 weeks), with or without 15 mM CaCl_2_ in drinking water	Male Wistar rats, 8 weeks old
[50]	↑ circulating 25D; vitamin D + FOS ↑ 1,25D, colonic VDR, TPH1 and serotonin; colonic Vdr positively correlated with Tph1	Females: vitamin D ↓ anxiety-like behavior in OF, with ↑ center time; Males: vitamin D alone no effect, vitamin D + FOS ↑ anxiety like behavior	Control (1000 IU/kg) or VD3-supplemented diet (5000 IU/kg), with or without 5% FOS and antibiotics	Male and female C57BL/6J mice, 6 weeks old
[31]	Predicted calcitriol targets related to anxiety/depression; involvement of neuronal signaling, neuroactive ligand–receptor interactions and JAK–STAT pathways	Not assessed; computational study only	Calcitriol used in network pharmacology and molecular docking analyses; no in vivo or in vitro administration	Not applicable (computational study)
[51]	↓ MDA, TNF-α and MAO-A; ↑ thiols, SOD, CAT, IL-10 and 5-HT	VD3 ↓ nicotine withdrawal-induced anxiety/depression-like behavior; ↑ center time in OFT; ↑ OA time in EPM; ↓ marble burying; ↓ immobility in FST	VD3 (100, 1000 or 10,000 IU/kg, i.p., 3-week nicotine withdrawal period after adolescent nicotine exposure (2 mg/kg)	Male Wistar rats, 60 days old
[52]	Improved HPA axis function; restored Bmal-1 expression; attenuated immune and gut–brain axis alterations	VD3 improved anxiety-like behavior and locomotion; ↑ center exploration in OFT; improved nest-building	VD3 1000 IU/kg after 28 days of sleep desynchrony	Male C57BL/6J mice, 5 weeks old
[53]	Hippocampal neuroprotection; ↓ serum TNF-α and IL-6	VD3 ↑ locomotor activity and exploration in OFT; ↑ open arm distance in EPM, indicating ↓ anxiety-like behavior, effects enhanced by exercise	VD3 (0.2 μg/kg/day, gavage, once daily for 8 weeks), with or without treadmill exercise	Male Sprague–Dawley rats, 8 weeks old

Abbreviations: ↑ = increase; ↓ = decrease; 5-HT = serotonin; 25(OH)D_3_ = 25-hydroxyvitamin D_3_; ACh = acetylcholine; ACT = activity cage test; CAT = catalase; DA = dopamine; DCX = doublecortin; DEX = dexamethasone; EPM = elevated plus maze; FOS = fructooligosaccharides; FST = forced swim test; HR = hole-board test; IL-6 = interleukin-6; LDT = light–dark test; MDA = malondialdehyde; NE = norepinephrine; OA = open arms; OF/OFT = open-field test; OVX = ovariectomy; PND = postnatal day; PNM = postnatal month; SOD = superoxide dismutase; TPH1 = tryptophan hydroxylase 1; UCMS = unpredictable chronic mild stress; VD_3_ = vitamin D_3_; VDR = vitamin D receptor; VdrKO = vitamin D receptor knockout; WT = wild-type.

The study by Kalueff et al. (2004) [36] investigated the role of vitamin Din brain function and emotional regulation by focusing on its nuclear receptor. Drawing on both human and animal evidence that links vitamin D dysfunction to behavioral disorders, the researchers explored the effects of genetically removing vitamin D receptors in mice. Their findings revealed that mice lacking these receptors exhibited increased anxiety-like behaviors across a range of behavioral tests. These results highlight the importance of vitamin D signaling in the brain and suggest that disruptions in its receptor activity may contribute significantly to alterations in emotional behavior, reinforcing the idea that vitamin D plays a crucial role in mental health.

Vitamin D has been increasingly associated with the modulation of anxiety-related behaviors, likely due to its role in brain function and neurogenesis. Evidence from animal studies suggests that vitamin D supplementation can exert anxiolytic effects, possibly by influencing hippocampal activity and promoting neuronal development. In particular, vitamin D may contribute to improved emotional regulation through its effects on neural plasticity and central signaling pathways. Supporting this notion, an experimental study in male Wistar rats showed that vitamin D supplementation reduced anxiety-like behaviors in the elevated plus maze, while also increasing hippocampal doublecortin expression, a marker of neurogenesis, suggesting a mechanistic link between vitamin D, neuronal development, and anxiety regulation (four-week supplementation study in male Wistar rats evaluating anxiety-related behavior and hippocampal neurogenesis) [49].

Data from rodent studies indicate that developmental vitamin D status plays a critical role in the regulation of anxiety-related behaviors. Maternal vitamin D deficiency in rats has been shown to increase anxiety- and depressive-like behaviors in adult offspring, accompanied by elevated pro-inflammatory cytokines and reduced prefrontal cortex expression of brain-derived neurotrophic factor (BDNF) and vitamin D receptor (VDR). These findings suggest that insufficient vitamin D during critical periods of brain development can disrupt neurotrophic signaling and neural circuitry involved in emotional regulation, predisposing offspring to long-lasting neuropsychiatric vulnerabilities. Importantly, interventions such as maternal vitamin D supplementation or treadmill exercise were able to prevent these behavioral and molecular alterations, highlighting the potential of vitamin D in mitigating anxiety-related outcomes through both direct effects on VDR-mediated pathways and indirect modulation of neuroinflammation (study in adult male rat offspring of vitamin D-deficient dams) [46].

Rodent studies demonstrate that gestational vitamin D supplementation can modulate anxiety-related behaviors in both dams and their offspring. vitamin D administration during pregnancy was reported to increase maternal care and produced an anxiolytic-like effect in the dams, although this effect was blocked when the mothers were treated with prenatal dexamethasone (DEX). Importantly, prenatal DEX exposure induced an anxiety-like phenotype in adult male and female offspring at six months of age, which was prevented by maternal vitamin D supplementation. These findings suggest that gestational vitamin D may protect against the development of anxiety-related behaviors in offspring, potentially through both direct neurodevelopmental effects and indirect improvements in maternal care [48].

The data presented by Ribeiro and MacDonald (2022) [45] establish a meaningful link between vitamin D and anxiety by showing that behavioral alterations in Mecp2-deficient mice, a model of Rett syndrome, correlate with circulating vitamin D levels. Specifically, mice with disrupted Mecp2 function exhibit anxiety-like behaviors alongside deficiencies in vitamin D and dysregulation of its synthesis pathway. Since vitamin D is known to inhibit NF-κB signaling, its supplementation helps restore molecular balance and improve neuronal structure. Importantly, this study demonstrates that improvements in anxiety-related behaviors occur in parallel with normalized vitamin D levels, suggesting that vitamin D plays a modulatory role in emotional regulation. These findings imply that vitamin D deficiency may exacerbate anxiety phenotypes, while its supplementation could help alleviate such symptoms by correcting underlying molecular and neuronal dysfunctions.

The study by Renteria et al. (2024) [50], conducted in C57BL/6J mice, reveals a complex and sex-specific relationship between vitamin D, gut microbiota, and anxiety-related behaviors. In female mice, vitamin D supplementation alone was shown to reduce anxiety-like behaviors, supporting the idea that adequate vitamin D levels can have a protective effect on emotional regulation. However, when combined with fructooligosaccharides (FOS), no additional benefit was observed, suggesting that gut microbiota modulation did not enhance this effect in females. In contrast, male mice displayed increased anxiety-like behaviors when given the combined vitamin D and FOS treatment, while vitamin D alone had no significant impact. The study also identified a positive correlation between colonic vitamin D receptor expression and TPH1, an enzyme critical for serotonin synthesis, indicating a potential mechanistic link between vitamin D signaling, gut function, and mood regulation. Overall, these results suggest that vitamin D influences anxiety through interactions with the gut–brain axis, but its effects vary depending on sex and microbiota-related factors.

A preclinical study in old ovariectomized rats demonstrated that chronic supplementation with vitamin D3 (cholecalciferol) at 1.0 mg/kg/day reduced anxiety-like behaviors, as assessed by the elevated plus maze, light–dark test, and open field test. The anxiolytic-like effects were further enhanced when vitamin D3 was combined with low-dose 17β-estradiol, indicating a synergistic interaction. Additionally, vitamin D3 treatment increased serum levels of 25-hydroxyvitamin D3 and estradiol compared to vehicle-treated controls, suggesting that its anxiolytic effects may be mediated in part by modulation of hormonal status. These findings support a beneficial role of vitamin D in alleviating anxiety-like behaviors in aged females with long-term estrogen deficiency [42].

A study in rats investigated the protective effects of vitamin D on anxiety-like behaviors induced by unpredictable chronic mild stress (UCMS). Vitamin D treatment reduced anxiety-like behaviors, as evidenced by improved performance in the elevated plus maze and open field tests. At the molecular level, UCMS increased markers of oxidative stress and neuroinflammation, including MDA and interleukin-6, while decreasing antioxidant enzymes such as CAT, SOD, and thiol. Vitamin D supplementation reversed these changes, suggesting that its anxiolytic effects are mediated, at least in part, by reducing brain oxidative stress and inhibiting neuroinflammatory processes. These findings support a neuroprotective role of vitamin D in stress-induced anxiety in rodents [43].

A study in male Wistar rats examined whether vitamin D3 could prevent anxiety-like behaviors induced by nicotine withdrawal. Vitamin D treatment effectively reduced anxiety-related behaviors in rats undergoing nicotine withdrawal, suggesting that vitamin D has anxiolytic potential in this model [51]. The study by Wu et al. (2021) [44] also demonstrated an anxiolytic effect of vitamin D3 in a nicotine withdrawal model. In a preclinical C57BL/6 mouse model of nicotine abstinence, dietary vitamin D3 supplementation reduced anxiety-like behaviors during withdrawal, as shown in open-field and marble-burying tests. This effect was accompanied by decreased hippocampal NR2A expression at both mRNA and protein levels, suggesting a possible mechanism involving modulation of glutamatergic signaling in the hippocampus.

A study in streptozotocin-induced type 1 diabetic rats demonstrated that vitamin D3 treatment reversed anxiety-like behaviors associated with diabetes. The anxiolytic effects of vitamin D3 were accompanied by regulation of purinergic signaling in the brain, including modulation of ectonucleotidase activity (NTPDase and 5′-nucleotidase), adenosine deaminase, and A1 and A2A receptor levels, suggesting that vitamin D3 may reduce diabetes-induced anxiety through neuroprotective mechanisms involving the purinergic system [46].

A preclinical study in rats investigated the impact of maternal dietary vitamin D_3_ levels on anxiety-related behaviors in the offspring. Both maternal vitamin D deficiency and excess increased anxiety-like behaviors in juvenile offspring, suggesting that deviations from optimal vitamin D status during early development can adversely affect emotional regulation. Notably, these behavioral alterations were restricted to the juvenile stage and were not observed in adulthood, highlighting a critical developmental window during which maternal vitamin D status shapes anxiety-related outcomes [38].

Another experimental study examined the effects of chronic cholecalciferol (vitamin D3) administration on anxiety-like behavior following long-term ovariectomy in female rats. Cholecalciferol at doses of 1.0 and 2.5 mg/kg, administered alone or in combination with low-dose 17β-estradiol, produced anxiolytic-like effects, as evidenced by increased open-arm exploration in the elevated plus maze, greater time in the light compartment in the light/dark test, and increased grooming activity in the open field test. These findings indicate that vitamin D3 can effectively reduce anxiety-like behaviors in a rat model of long-term estrogen deficiency [39]. Another study conducted in ovariectomized female rats examined the effects of chronic high-dose cholecalciferol (vitamin D3) administration on anxiety-like behavior. Cholecalciferol, given alone or in combination with 17β-estradiol, produced anxiolytic-like effects, as evidenced by increased open-arm exploration in the elevated plus maze, greater time in the light compartment in the light–dark test, and increased grooming activity in the open field test. These behavioral effects were accompanied by elevated hippocampal dopamine and serotonin levels and reduced serotonin turnover, suggesting that the anxiolytic-like effects of vitamin D3 may be mediated, at least in part, through modulation of monoaminergic signaling in the hippocampus [40].

A study in Sprague-Dawley rats investigated the effects of vitamin D supplementation, physical exercise, and their combination on anxiety-like behaviors induced by chronic unpredictable mild stress (CUMS). Vitamin D alone, physical exercise alone, and particularly the combination of both, alleviated anxiety-like behaviors, as evidenced by increased exploratory activity in the open field test and greater open-arm exploration in the elevated plus maze. Biochemically, these interventions reduced serum levels of the pro-inflammatory cytokines TNF-α and IL-6 and protected hippocampal neurons from stress-induced damage, suggesting that the anxiolytic effects of vitamin D may be mediated, at least in part, through anti-inflammatory and neuroprotective mechanisms [53].

A study investigating the effects of vitamin D_3_ supplementation in mice subjected to chronic sleep desynchrony found that vitamin D_3_ supplementation partially improved behavioral alterations associated with circadian disruption. The treated animals exhibited increased locomotor and exploratory activity, as well as reduced anxiety-like behavior. These effects were accompanied by improvements in hypothalamic–pituitary–adrenal (HPA) axis function, restoration of Bmal-1 expression, and attenuation of immune and gut–brain axis alterations induced by sleep desynchrony. While the role of vitamin D_3_ in anxiety regulation appears limited, these results imply that vitamin D could promote behavioral resilience under chronic circadian stress via neuroendocrine and immunological mechanisms [52].

Although many studies suggest an association between vitamin D supplementation and anxiolytic-like effects, some studies have not demonstrated a relationship between vitamin D status and anxiety-related behaviors. The study by Kalueff et al. (2004) [37] shows that mice lacking vitamin D receptors exhibit increased grooming behavior, but this change did not correlate with anxiety-like behaviors, as measures from tests such as the elevated plus maze and hole board remained unaltered. Importantly, this study examined adult male mice with a constitutive genetic ablation of VDR, rather than vitamin D deficiency or supplementation. Thus, the absence of an anxiety-like phenotype in this model should not necessarily be interpreted as evidence that vitamin D signaling is unrelated to anxiety. Rather, it suggests that disruption of VDR signaling may affect specific behavioral domains, such as grooming, without producing detectable changes in anxiety-like behavior under the experimental conditions used. In addition, Liang et al. (2018) [41] found that postnatal vitamin D deficiency or supplementation did not significantly alter anxiety- or depression-like behaviors in C57BL/6 mice. In this study, animals were exposed postnatally to diets containing 0, 1000, or 10,000 IU vitamin D3, representing deficiency, standard intake, and overdose, respectively. Interestingly, although anxiety-like behavior was unaffected, abnormal vitamin D intake was associated with other behavioral and cognitive consequences, including transient motor effects and impairments in spatial learning and long-term memory. Taken together, these studies suggest that the relationship between vitamin D signaling and anxiety-like behavior is unlikely to be strictly linear or consistent across experimental conditions. In particular, the findings of Liang et al. [41] raise the possibility that both insufficient and excessive vitamin D exposure may disrupt neural homeostasis, whereas the VDR knockout findings [37] indicate that disruption of receptor signaling may produce behavioral phenotypes that seem unrelated to anxiety.

## 4. Integrating Preclinical Evidence with Network Pharmacology

**Figure 1:**
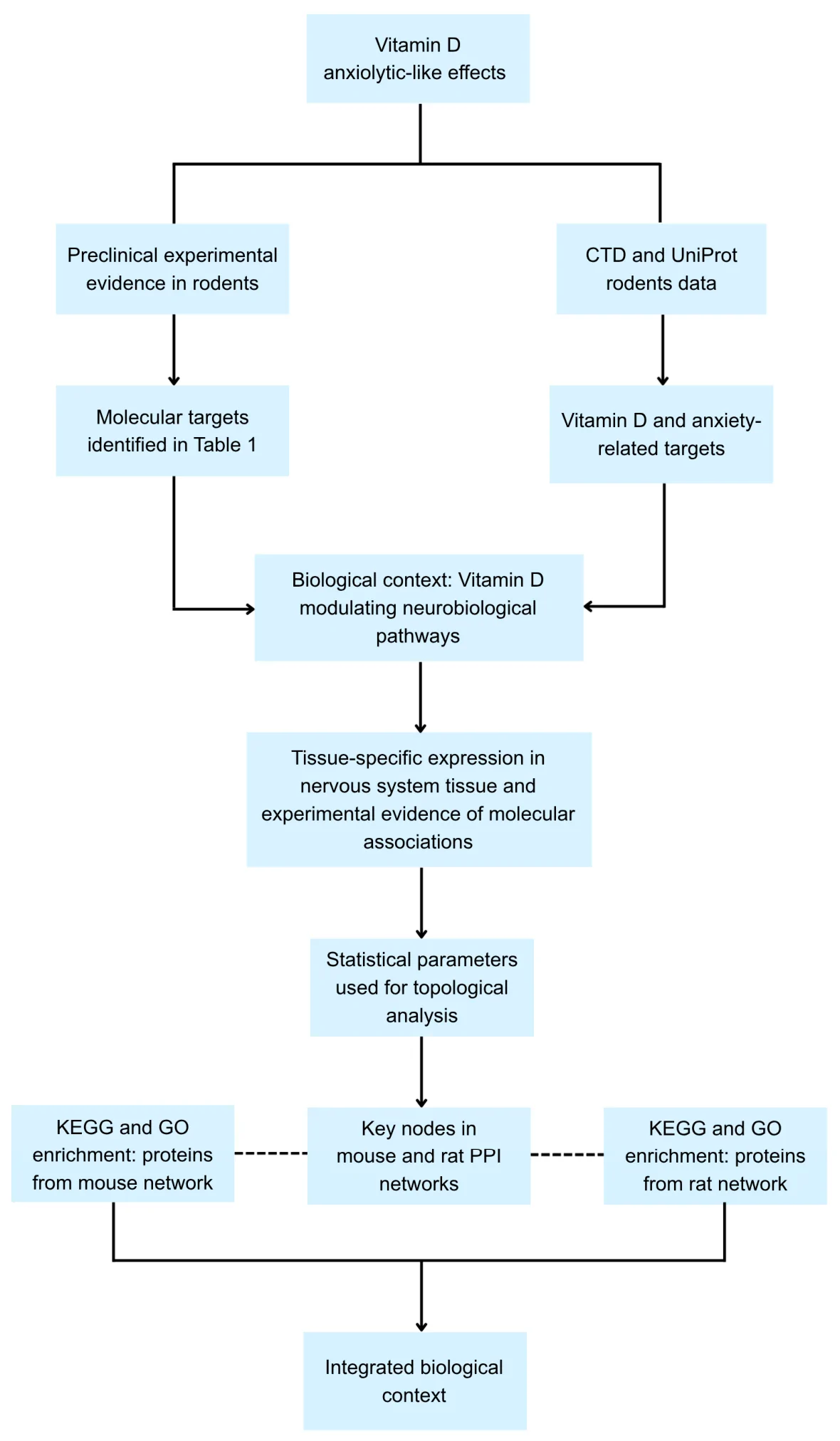
Preclinical–bioinformatics integration framework. Integration of the preclinical evidence summarized in Table 1 with the molecular targets identified through searches of the CTD and UniProt databases. Protein–protein interactions identified through STRING-based network analysis were interpreted in the context of the biological effects observed in preclinical studies. This framework links the predicted molecular interactions and enriched biological pathways with the observed phenotypic outcomes, providing a basis for generating potential mechanistic hypotheses.

Although preclinical studies suggest anxiolytic-like effects of vitamin D3 in rodent models, the molecular mechanisms potentially underlying these effects have not been fully elucidated. As previously mentioned in this review, experimental evidence indicates that vitamin D3 may influence multiple biological processes associated with anxiety-related behavior, including neurotransmission, neuroplasticity, neuroinflammation, and oxidative stress. These processes provide a biological context for interpreting the molecular targets and signaling pathways identified through network pharmacology. However, the mechanisms involved have generally been investigated across different experimental models and biological levels, making it difficult to obtain an integrated view of the molecular networks potentially linking vitamin D3 to anxiety-related behavior. In this context, network pharmacology and bioinformatic approaches can complement the preclinical evidence by identifying potential molecular targets, biological pathways, and functional interactions that may converge on these experimentally observed processes. Therefore, to complement the experimental evidence summarized in this review, we performed a network-based bioinformatic analysis to explore molecular targets and signaling pathways potentially associated with the effects of vitamin D3 on anxiety-related behavior. The resulting computational predictions were subsequently considered in relation to the preclinical findings, with the aim of identifying areas of convergence and generating mechanistic hypotheses for future experimental validation. The relationship between the preclinical evidence and the network pharmacology analysis is summarized in an integrated framework diagram (Figure 1).

Unlike previous bioinformatic studies focusing on calcitriol [31], the present analysis is centered on vitamin D3 (cholecalciferol), the form most administered in rodent models of anxiety, allowing a closer integration between network pharmacology predictions and the available preclinical evidence.

The CTD search identified 2873 vitamin D3-related targets and 62 anxiety-related targets. After restricting the anxiety dataset to curated records associated with DSM-5 anxiety disorders, 53 targets remained. In UniProt, 37 anxiety-related targets and 119 vitamin D3-related targets were identified for *Mus musculus*, whereas 37 anxiety-related targets and 29 vitamin D3-related targets were identified for *Rattus norvegicus*. The overlap analysis identified 14 shared targets between anxiety and vitamin D3 in the CTD, namely: Fos (Protein c-Fos), Tnf (Tumor necrosis factor), Drd2 (D2 dopamine receptor), Slc6a3 (Sodium-dependent dopamine transporter), Dnmt1 (DNA methyltransferase 1), Mapt (Microtubule-associated protein tau), Crp (C-reactive protein), Tg (Thyroglobulin), Mdk (Midkine), Serpina1 (Plasminogen activator inhibitor 1), Eomes (Eomesodermin), Ins (Insulin), Ifna2 (Interferon alpha-2) and Cat (Catalase). Only one shared target, Bglap (Osteocalcin) was identified by intersection of the UniProt data. The proteins identified by STRING analysis are presented in Supplementary Table S1.

**Figure 2:**
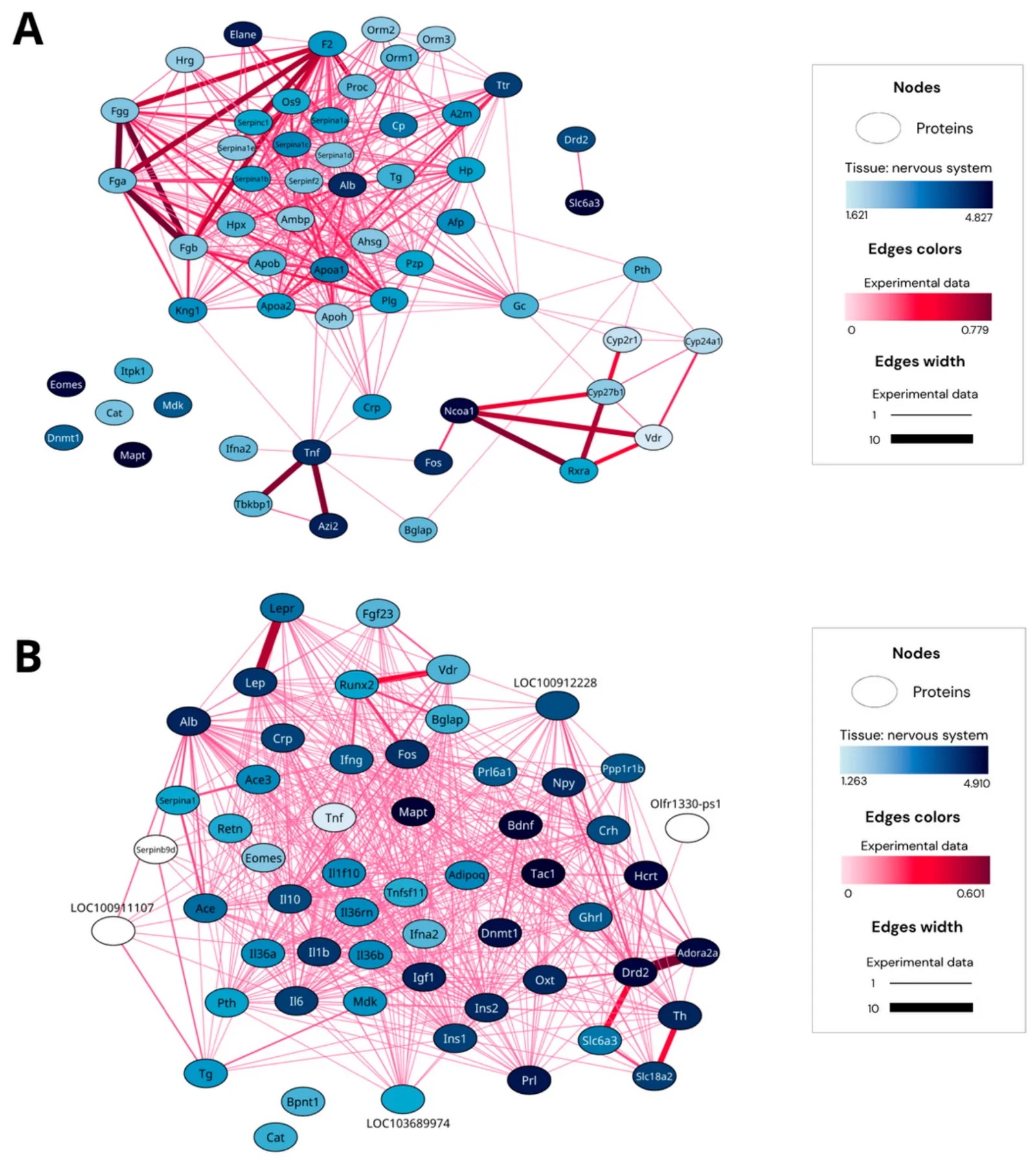
Protein–protein interaction (PPI) networks among vitamin D3 and anxiety molecular targets in rodents. (**A**) Mouse PPI network composed of 58 nodes and 391 edges. (**B**) Rat PPI network composed of 56 nodes and 603 edges. Both networks’ statistical parameters were based on the target’s presence in nervous system (NS) tissue, according to the STRING score range of 1.0–5.0. Darker node colors (blue) indicate a stronger presence in NS tissue. The edge color gradient (red) and width refer to the amount of experimental evidence according to the STRING score range of 0.0–1.0 and 1.0–10.0, respectively.

Cross-referencing confirmed the presence of the 14 CTD-derived shared targets in both *Mus musculus* and *Rattus norvegicus*. After manual inclusion of VDR, a total of 16 molecular targets were used as input for STRING. For the mouse network, STRING added two additional interacting proteins (Serpina1a and Serpina1d), resulting in 18 input proteins. However, some of the protein identifiers were not directly recognized for the species of interest in the STRING database. In these cases, the platform provided corresponding or related protein entries based on available database annotations. These mapped entries were subsequently considered for network construction and functional enrichment analysis. The final mouse PPI network comprised 58 nodes and 391 edges, whereas the rat PPI network comprised 56 nodes and 603 edges (Figure 2).

Of the proteins described in the literature as potential molecular targets that mediate the anxiolytic effects of vitamin D, and which are summarized in Table 1, the rat STRING network also identified the following: adenosine A2A receptor, BDNF, catalase, dopamine D2 receptor, interleukin-10, interleukin-6, tumor necrosis factor-α (TNF-α) and vitamin D receptor (VDR). The mouse STRING network identified the following proteins reported in the literature as potential molecular targets underlying the anxiolytic effects of vitamin D (Table 1): catalase, CYP24A1, CYP27B1, dopamine D2 receptor, interleukin-10, interleukin-6, tumor necrosis factor-α (TNF-α) and vitamin D receptor (VDR). Overall, the molecular targets identified in the mouse and rat networks were largely comparable, with only minor differences. These discrepancies, including the absence of BDNF and the identification of CYP24A1 and CYP27B1 in the mouse network, are more likely related to differences in database annotation and coverage than to genuine biological differences between the two species.

While Table 1 summarizes the molecular targets reported in experimental studies showing anxiolytic effects of vitamin D in mice and rats, the STRING analysis further identified additional proteins, including interferons, IGF-1, IL-1, Slc18a2 (rat network), and CYP2R1 (mouse network). These findings suggest that the molecular mechanisms potentially involved in the anxiolytic effects of vitamin D may extend beyond those previously described in the experimental literature, particularly by reinforcing the potential involvement of neuroinflammatory pathways, monoaminergic neurotransmission, and vitamin D metabolism.

Furthermore, the convergence networks between anxiety and vitamin D identified several additional proteins with established roles in central nervous system function, including albumin, DNA (cytosine-5)-methyltransferase (DNMT), orexin A, leptin, microtubule-associated tau protein, prolactin, dopamine transporter (DAT), nuclear receptor coactivator 1 (NCOA1), and transthyretin (TTR). These proteins are involved in processes such as epigenetic regulation, neuroendocrine signaling, monoaminergic neurotransmission, energy homeostasis, neurodegeneration, and synaptic function, suggesting additional biological pathways through which vitamin D may influence anxiety-related behaviors [54,55,56,57,58,59,60,61,62,63].

**Figure 3:**
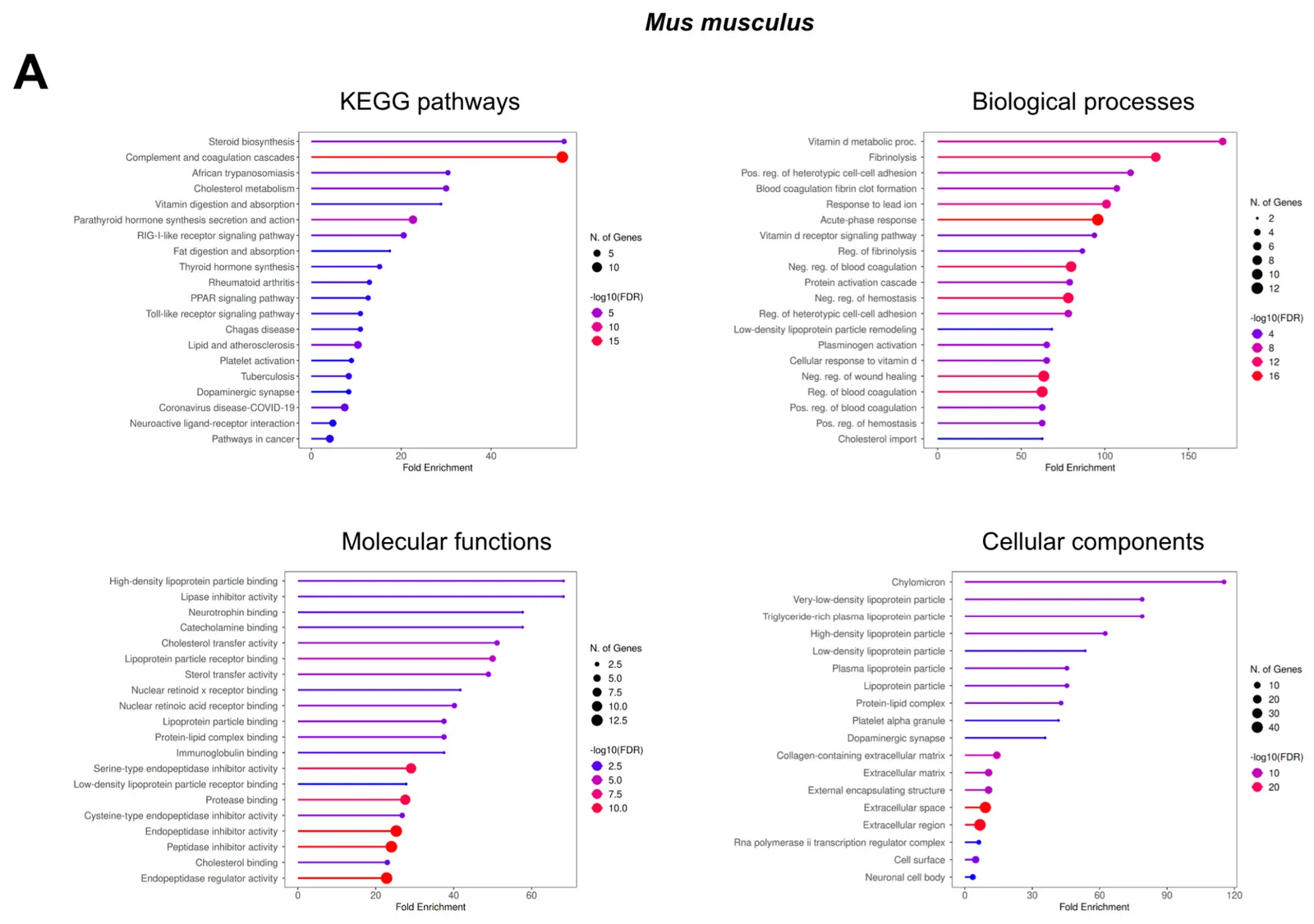

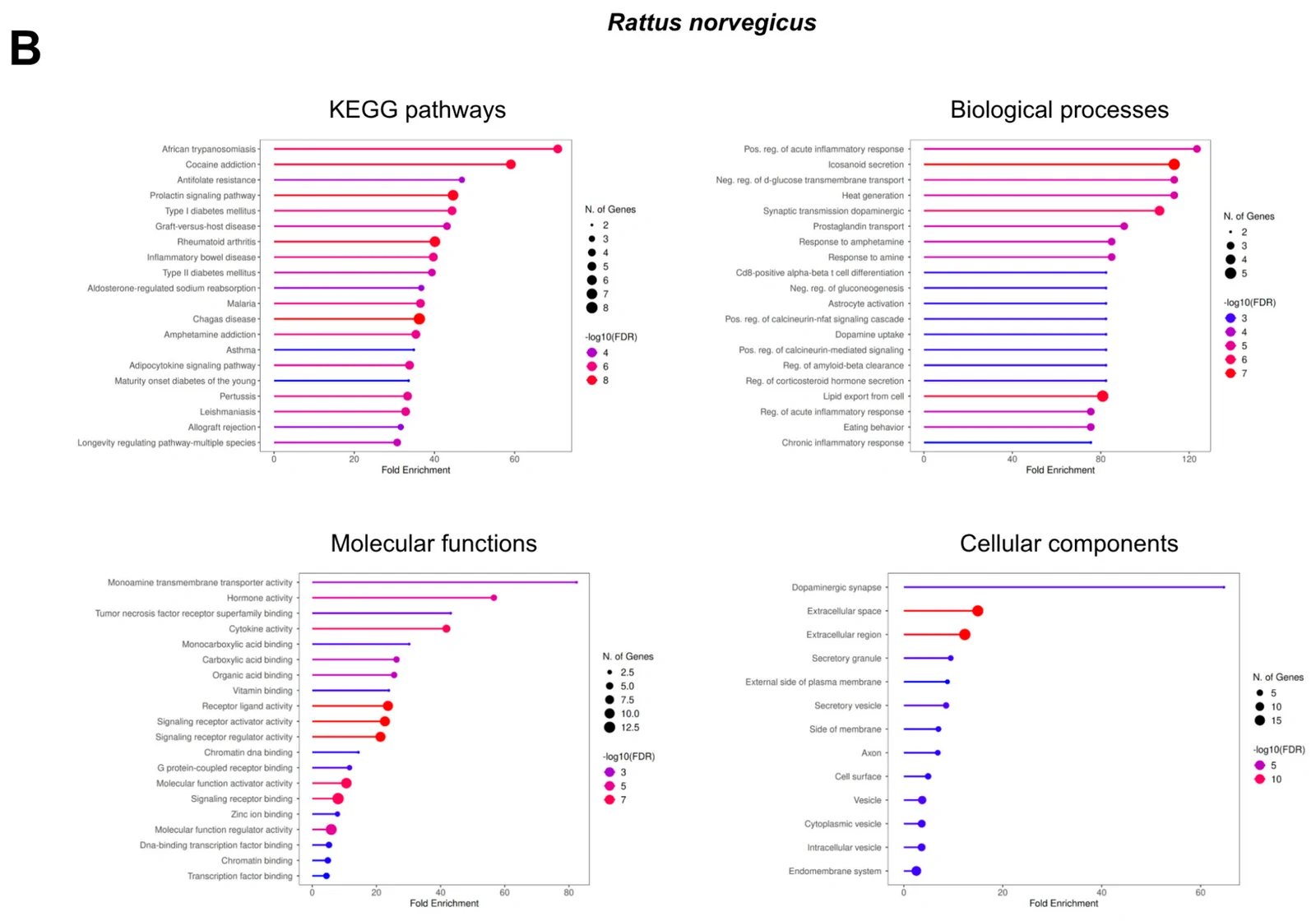
GO and KEGG enrichment analysis of vitamin D3- and anxiety-related molecular targets from mouse and rat PPI networks constructed using STRING. (**A**) KEGG pathways and GO terms for biological processes, molecular functions, and cellular components identified from molecular targets in the mouse PPI network. (**B**) KEGG pathways and GO terms for biological processes, molecular functions, and cellular components identified from molecular targets in the rat PPI network. Fold enrichment is shown on the x-axis. The −log10(FDR) values are represented by color, with colors closer to red indicating greater statistical significance.

To further characterize the biological processes associated with these network-derived targets, GO and KEGG enrichment analyses were performed. The complete GO and KEGG enrichment results are presented in Figure 3. The functional enrichment analysis identified several biological processes and signaling pathways potentially relevant to the effects of vitamin D3 on anxiety-related behavior. In mice, the enriched terms included vitamin D metabolic processes, acute-phase response, complement and coagulation cascades, parathyroid hormone signaling, and steroid biosynthesis, suggesting potential involvement of vitamin D-related metabolic, hormonal, and inflammatory processes. In rats, enrichment of dopaminergic synaptic transmission, dopaminergic synapse, monoamine transmembrane transporter activity, cytokine activity, and eicosanoid-related processes highlighted potential links between neurochemical signaling and inflammatory pathways. Taken together, these findings indicate that the molecular targets identified across the preclinical models may converge on metabolic, inflammatory, hormonal, and monoaminergic processes, providing potential biological context for the behavioral effects observed in experimental studies. However, these enrichment results should be considered hypothesis-generating and require experimental validation to establish their specific contribution to the effects of vitamin D3 on anxiety-related behavior.

**Figure 4:**
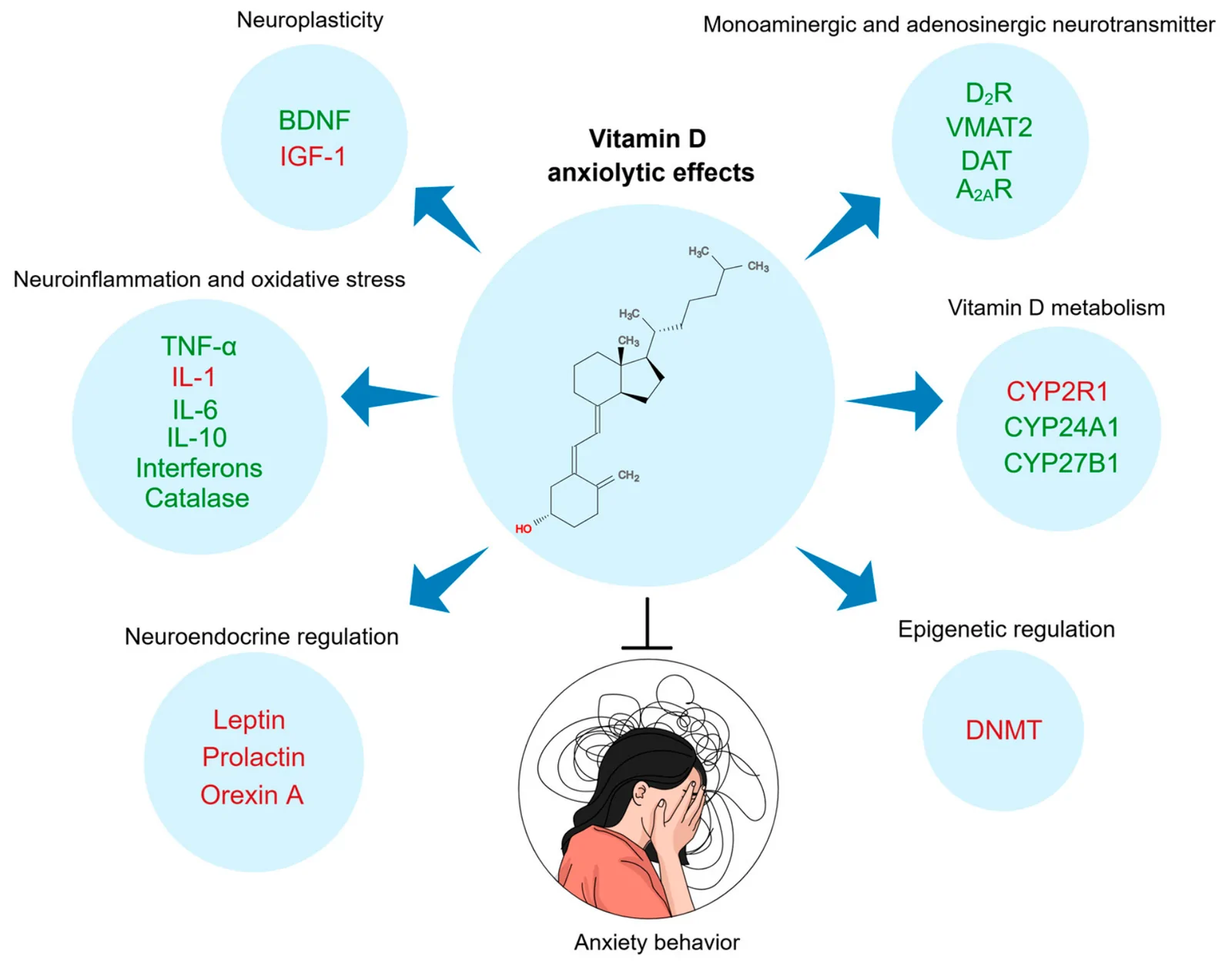
Mechanisms potentially involved in the anxiolytic-like effect of vitamin D. Molecular targets found in both Table 1 and the protein–protein interaction (PPI) networks for mice and rats constructed in STRING are represented in green. Proteins found in the mice and rats PPI networks but not in Table 1 are represented in red.

Collectively, as illustrated in Figure 4 our results suggest that the potential anxiolytic effects of vitamin D may involve the coordinated modulation of multiple biological processes, including neuroinflammatory signaling, neuroplasticity, monoaminergic neurotransmission, neuroendocrine regulation, epigenetic mechanisms, and vitamin D metabolism, all of which have been implicated in the pathophysiology of anxiety disorders [3,4,12,17,64,65]. Notably, several of these processes overlap with biological mechanisms identified in the preclinical studies, providing potential points of convergence between the experimental and network pharmacology findings. However, the molecular associations identified through the bioinformatic analysis should be considered hypothesis-generating and do not establish causal mechanisms. Experimental validation will be necessary to determine whether these predicted targets and pathways contribute functionally to vitamin D-associated modulation of anxiety-related behavior.

## 5. Conclusions

Together, preclinical findings support the hypothesis that vitamin D status may influence anxiety-related behavior through multiple, potentially convergent biological mechanisms. These findings provide a basis for further investigation of the role of vitamin D in anxiety-related processes; however, their relevance to human anxiety disorders and their therapeutic efficacy remain to be established. Future studies integrating clinical outcomes with mechanistic biomarkers will be important to determine whether the pathways identified in preclinical models are also relevant in humans and whether vitamin D supplementation may have clinically meaningful effects, particularly in individuals with vitamin D deficiency. Such studies may also help clarify appropriate dosing strategies, therapeutic thresholds, and treatment windows, if a clinically relevant effect is confirmed.

Overall, further mechanistic, preclinical, and clinical research is warranted to better characterize the molecular pathways through which vitamin D may influence neuroinflammation, neuroplasticity, oxidative stress, and neurotransmitter-mediated signaling, and to determine whether these mechanisms translate into clinically meaningful effects on anxiety-related outcomes. In addition, expanding bioinformatics-based approaches and experimentally validating their predictions will be important to generate and test new mechanistic hypotheses, identify potential molecular targets, and strengthen the translational relevance of vitamin D research in anxiety-related disorders.

Translating the findings from rodent models to clinical research requires consideration of several factors that may limit direct extrapolation, including interspecies differences in vitamin D metabolism, pharmacokinetics, dose exposure, and VDR expression. Therefore, doses that produce behavioral effects in experimental models should not be directly extrapolated to humans, and future clinical studies should be guided by established human pharmacokinetic and safety data. Patient selection may also be important, particularly regarding baseline vitamin D status and biological characteristics that may influence vitamin D signaling. Populations with vitamin D deficiency or insufficiency may therefore represent a reasonable starting point for clinical investigation, while the potential relevance of inflammatory, stress-related, or hormonal factors should be evaluated prospectively rather than assumed. Future randomized controlled trials should incorporate baseline vitamin D status, standardized measures of anxiety-related symptoms, and, where feasible, relevant biological markers to determine whether vitamin D supplementation produces clinically meaningful effects and whether any observed effects are associated with specific biological profiles.

## Data Availability

The original contributions presented in this study are included in the article. Further inquiries can be directed to the corresponding author.

## Supplementary Materials

The following supporting information can be downloaded at: https://www.mdpi.com/article/10.3390/nu18162707/s1, Table S1: Additional proteins identified by STRING analyses for each species.

## Abbreviations

The following abbreviations are used in this manuscript:

1,25-D_3_	1,25-dihydroxyvitamin D_3_ (calcitriol)
5-HT	5-hydroxytryptamine (serotonin)
8-OHdG	8-hydroxy-2′-deoxyguanosine
BDNF	brain-derived neurotrophic factor
Bmal-1	brain and muscle ARNT-like protein 1
CAT	catalase
CUMS	chronic unpredictable mild stress
DA	dopamine
DAT	dopamine transporter
DEX	dexamethasone
DNMT	DNA methyltransferase
DSM-5	Diagnostic and Statistical Manual of Mental Disorders, Fifth Edition
FDR	false discovery rate
FOS	fructooligosaccharides
GABA	gamma-aminobutyric acid
GO	gene ontology
HPA	hypothalamic–pituitary–adrenal
IGF-1	insulin-like growth factor 1
IL-1	interleukin-1
IL-6	interleukin-6
IL-10	interleukin-10
KEGG	Kyoto Encyclopedia of Genes and Genomes
MAO-B	monoamine oxidase B
MDA	malondialdehyde
MetS	metabolic syndrome
NCOA1	nuclear receptor coactivator 1
NE	norepinephrine
NF-κB	nuclear factor kappa B
NKAα1	Na^+^/K^+^-ATPase alpha 1
NLRP3	NLR family pyrin domain-containing 3
NR2A	N-methyl-D-aspartate receptor subunit 2A
NTPDase	nucleoside triphosphate diphosphohydrolase
P2X7R	P2X7 receptor
PPI	protein–protein interaction
SNRIs	serotonin–norepinephrine reuptake inhibitors
SOD	superoxide dismutase
SSRIs	selective serotonin reuptake inhibitors
TNF-α	tumor necrosis factor alpha
TPH1	tryptophan hydroxylase 1
TTR	transthyretin
UCMS	unpredictable chronic mild stress
VDR	vitamin D receptor
