# Supplementary material for: Neurobiological Mechanisms of Vitamin D in Anxiety: Evidence from Rodent Models and Bioinformatics-Based Target Identification
Source: Nutrients. 2026 Aug 19;18(16):2707. doi: 10.3390/nu18162707 (PMC13516192; doi:10.3390/nu18162707)
Supplement: Supplementary file 1 [file nutrients-18-02707-s001.zip › nutrients-4502254-supplementary.pdf]

Supplementary Table S1. Additional proteins identified by STRING analyses for each species.

Additional proteins (mouse PPI network)	Additional proteins (rat PPI network)
A2m (Alpha-2-macroglobulin)	Ace (Angiotensin-converting enzyme, soluble form)
Afp (Alpha-fetoprotein)	Ace3 (Angiotensin-converting enzyme)
Ahsg (Alpha-2-HS-glycoprotein)	Adipoq (Adiponectin)
Alb (Albumin)	Adora2a (Adenosine receptor A2a)
Ambp (Protein AMBP, Alpha-1-microglobulin)	Alb (Albumin)
Apoa1 (Apolipoprotein A-I)	Bdnf (Brain-derived neurotrophic factor)
Apoa2 (Apolipoprotein A-II)	Bpnt1 (3'(2'),5'-bisphosphate nucleotidase 1)
Apob (Apolipoprotein B-100)	Crh (Corticoliberin)
Apoh (Beta-2-glycoprotein 1)	Fgf23 (Fibroblast growth factor 23)
Azi2 (5-azacytidine-induced protein 2)	Ghrl (Appetite-regulating hormone)
Cp (Ceruloplasmin)	Hcrt (Orexin-A, Hypocretin neuropeptide precursor)
Cyp24a1 (1,25-dihydroxyvitamin D(3) 24-hydroxylase, mitochondrial)	Ifng (Interferon gamma)
Cyp27b1 (25-hydroxyvitamin D-1 alpha hydroxylase, mitochondrial)	Igf1 (Insulin-like growth factor I)
Cyp2r1 (Vitamin D 25-hydroxylase)	Il1b (Interleukin-1 beta)
Elane (Neutrophil elastase)	Il1f10 (Interleukin-1 family member 10)
F2 (Activation peptide fragment 1, Prothrombin)	Il6 (Interleukin-6)
Fga (Fibrinogen alpha chain)	Il10 (Interleukin-10)
Fgb (Fibrinogen beta chain)	Il36a (Interleukin-1)
Fgg (Fibrinogen gamma chain)	Il36b (Interleukin-1)
Gc (Vitamin D-binding protein)	Il36rn (Interleukin-1)
Hp (Haptoglobin)	Ins1 (Insulin-1)
Hpx (Hemopexin)	Ins2 (Insulin-2)
Hrg (Histidine-rich glycoprotein)	Lep (Leptin)
Itpk1 (Inositol-tetrakisphosphate 1-kinase)	Lepr (Leptin receptor)
Kng1 (Kininogen-1 heavy chain)	LOC100911107 (Leukocyte elastase inhibitor A-like)
Ncoa1 (Nuclear receptor coactivator 1)	LOC100912228 (Neuropeptide Y)
Orm1 (Alpha-1-acid glycoprotein 1)	LOC103689974 (Tumor necrosis factor ligand superfamily member 11, membrane form)
Orm2 (Alpha-1-acid glycoprotein 2)	Npy (C-flanking peptide of NPY, Pro-neuropeptide Y)
Orm3 (Alpha-1-acid glycoprotein 3)	Olfr1330-ps1 (Olfactory receptor 1330, pseudogene 1)
Os9 (Protein OS-9)	Oxt (Oxytocin-neurophysin 1)
Plg (Plasmin, Plasminogen)	Ppp1r1b (Protein phosphatase 1 regulatory subunit 1B)

Proc (Vitamin K-dependent protein C)	Prl (Prolactin)
Pth.(Parathyroid hormone)	Prl6a1 (Prolactin-6A1)
Pzp (Alpha-2-macroglobulin 165 kDa subunit)	Pth.(Parathyroid hormone)
Rxra (Retinoic acid receptor RXR-alpha)	Retn (Resistin)
Serpina1b (Alpha-1-antitrypsin 1-2)	Runx2 (Runt-related transcription factor 2)
Serpina1c (Alpha-1-antitrypsin 1-3)	Serpinb9d (Serine (or cysteine) proteinase inhibitor, clade B, member 9e)
Serpina1e (Alpha-1-antitrypsin 1-5)	Slc18a2 (Synaptic vesicular amine transporter)
Serpinc1 (Antithrombin-III)	Tac1 (C-terminal-flanking peptide, Tachykinin,1)
Serpinf2 (Alpha-2-antiplasmin)	Th (Tyrosine 3-monooxygenase)
Tbkbp1 (TANK-binding kinase 1-binding protein 1)	Tnfsf11 (TNF superfamily member 11)
Ttr (Transthyretin)	-
